# Peritoneal dialysis catheter malfunction caused by wrapping of the catheter by the sigmoid mesocolon: a case report

**DOI:** 10.1080/0886022X.2021.1879854

**Published:** 2021-02-04

**Authors:** Xiao-Jiao Shao, Li Yuan Zhang

**Affiliations:** The Affiliated Lianyungang Hospital of Xuzhou Medical University, Nephrology, China

Editor,

A 45-year-old man was admitted in our hospital with high serum creatinine level (522.3 μmol/L). Abdominal ultrasound demonstrated symmetrically reduced kidney size. With the history of hypertension, anemia, and abnormalities of calcium and phosphorus, phosphorus, the patient was diagnosed with chronic renal failure secondary to chronic glomerulonephritis. Coiled peritoneal dialysis (PD) catheter was placed under laparoscopic guidance and general anesthesia on May 17, 2019. The free greater omentum in the pelvic cavity was observed during the operation, which was folded and fixed in the upper abdominal area with Hem-o-lok clips. Continuous ambulatory peritoneal dialysis (CAPD) was performed with PD fluid containing 1.5% dextrose, 3 times/day. The dwell volume was 0.5 L. After three days, the dose was changed to 1 L, 3 times/day. The patient was readmitted to our hospital for the low outflow of PD fluid on May 29, 2019. A plain abdominal film showed that the PD catheter was still in the optimal position; therefore, the catheter obstruction was suspected. A diagnostic laparoscopy was performed on June 5, 2019. The operative finding revealed that the PD catheter was wrapped by the sigmoid mesocolon (Figure 1). The tissue collected from the PD catheter lumen was sent for a pathological examination (Figure 2). Subsequently, the PD catheter was exposed, and anti-adhesion drugs (sodium hyaluronate 3 mL) were applied locally. Both inflow and outflow rates were satisfied after the operation. However, the catheter was obstructed again as no fluid could be instilled and drained on 11 June 2019, which was accompanied by abdominal pain. The PD catheter was subsequently removed, and the patient was switched to hemodialysis. The patient has good symptoms and is being followed up.

**Figure 1.**
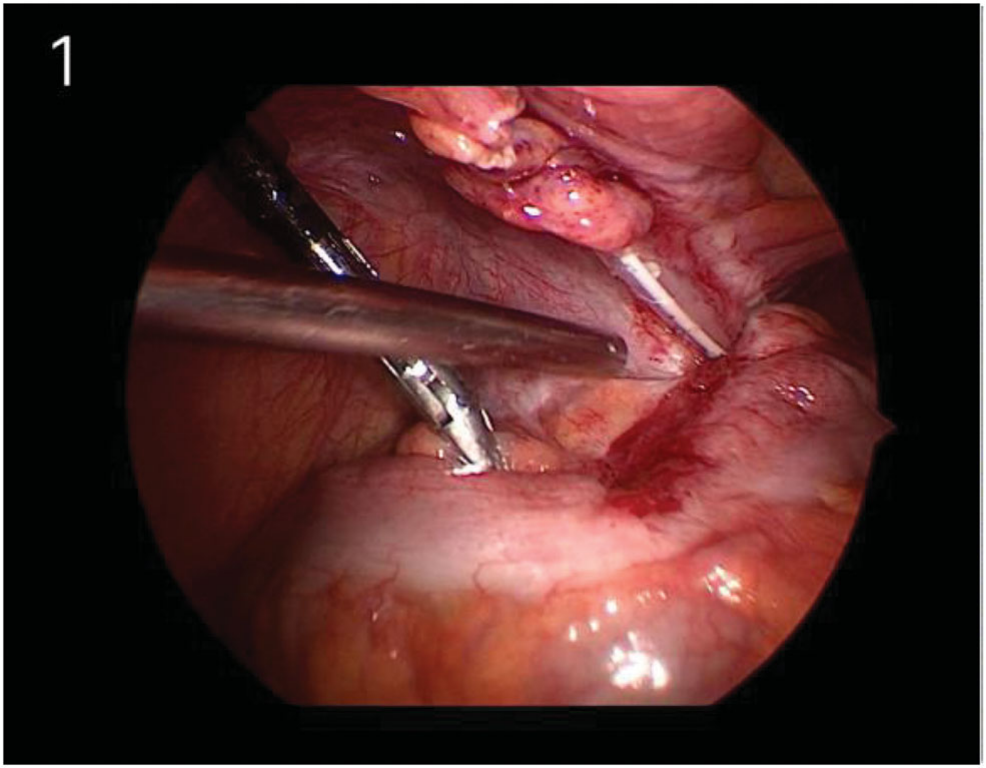
The PD catheter (Tenckhoff catheter) was wrapped by the sigmoid mesocolon.

**Figure 2.**
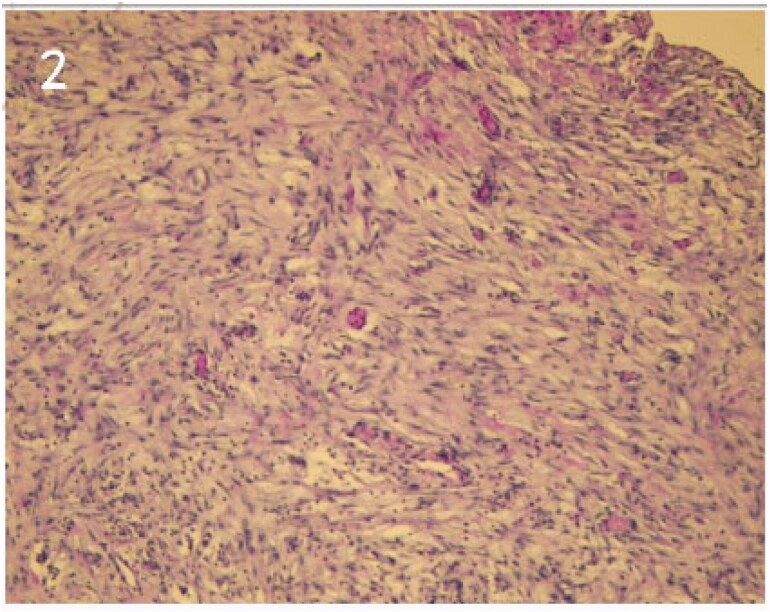
The tissue coming from the PDcatheter lumen showed exudation of sigmoid mesocolon tissue.

## Discussion

Complications associated with PD are generally classified as infectious and noninfectious [1]. Catheter malfunction is the most common cause of the latter. We first used abdominal radiography in this patient to exclude the most common causes of catheter malfunction, including intraluminal thrombosis, dislocation of the catheter tip, and catheter kinking. Plain radiographic imaging confirmed a dialysis catheter with the tip located in the pelvis. We also excluded PD catheter leak, which usually occurs in the first weeks after insertion. No signs of asymmetrical subcutaneous swelling and edema were observed. The patient was treated with PD catheterization, where the omentum was folded and fixed with Hem-o-lok clips under laparoscopy surgery. The advantage of our technique is that the incidence of postoperative complications such as catheter displacement and leakage of omentum wrap are minimal [2]. When both outflow and inflow failures are observed, especially in conjunction with abdominal pain, the possibility of obstruction by the abdominal viscera, including the wrap of omentum [3,4], hemorrhagic corpus luteum [5], the fimbriae and infundibulum of the oviduct [6], fallopian tube [7], fibrin sheath [1], appendix [8], small intestine [9], sigmoid colon wall [10]. Omental capture was suspected, and diagnostic laparoscopy was performed.The omentum was not involved,but the sigmoid mesocolon tightly covered the catheter and blocked the drainage holes.

To the best of our knowledge, this is the first case which reported that sigmoid mesocolon is the cause of complete obstruction of a Tenckhoff catheter. There was no history of constipation or vomiting. The patient has a bad habit of discharging fluid for a long time. We found that the patient had a large sigmoid mesocolon observed by the surgical video. We hypothesize that, under the influence of the negative pressure of outflow, the sigmoid mesocolon was pulled toward the external wall of the catheter, occluding the side ports and thus preventing dialysate outflow. The anti-adhesion drugs were applied locally in the diagnostic laparoscopy, and the free sigmoid mesocolon was fixed with Hem-o-lok clips. Unfortunately, the catheter failed again. This case shows that when the PD catheter malfunction is encountered, one should not ignore the possibility of obstruction by the sigmoid mesocolon.
